# Health economic evaluation of EV71 vaccination in children under the school-age population in Guangdong Province, China

**DOI:** 10.3389/fpubh.2025.1514501

**Published:** 2025-05-22

**Authors:** Hui Zhang, Jienan Zheng, Danxia Luo, Biao Zeng, Yao Yi, Fen Yang, Lilin Lin, Aiping Deng, Min Kang, Yingtao Zhang

**Affiliations:** ^1^School of Medical Business, Guangdong Pharmaceutical University, Guangzhou, Guangdong, China; ^2^Guangdong Health Economics and Health Promotion Research Center, Guangzhou, Guangdong, China; ^3^Guangdong Provincial Center for Disease Control and Prevention, Guangzhou, China

**Keywords:** hand, foot and mouth disease, EV71 vaccine, health economics evaluation, vaccine, EV71

## Abstract

**Background:**

This study aims to evaluate the health economics of hand-foot-mouth disease enterovirus 71 (EV71) vaccination for the population of appropriate age in Guangdong Province.

**Methods:**

A SEIR model was constructed, and a group of differential equations was established. The incidence data of HFMD in Guangdong from January to June 2017 were used to fit the model and the basic reproduction value (R_0_) of this disease was simulated. Then, the incidence of HFMD under different vaccination coverage rate (0, 40, 70, and 90%) was simulated in four scenarios. Cost-effectiveness analysis was used to evaluate the health economics.

**Results:**

The self-funded voluntary EV71 vaccination strategy implemented in Guangdong Province has effectively reduced the disease economic burden of EV71-type HFMD, and the disease economic burden saved during the peak seasonal segment of HFMD in 2017 was $1,080,000. Meanwhile, Scenario 2, 3, and 4 would each result in a cumulative reduction of 6,525, 9,556, and 10,989 confirmed cases, respectively, with net monetary benefits of approximately $6.55 million, $9.59 million, and $11.2 million. The study results show that the current vaccine pricing is not cost-effectiveness, while the vaccine price is lower than $13.15, EV71 vaccination in Guangdong Province has a cost-effectiveness advantage.

**Conclusion:**

Vaccination can reduce the incidence of HFMD caused by EV71, which helps to improve the status of HFMD and decreases the disease burden.

## 1 Introduction

Hand, foot and mouth disease (HFMD) is a common infectious disease caused by a variety of human enteroviral infections, which is most common in children under 5 years of age (1). The main symptoms of the disease are characterized by maculopapular and herpes eruptions on the palms, soles, oral mucosa and buttocks, with some patients experiencing serious complications. The majority of patients have mild symptoms that resolve spontaneously, while a small number of severely ill patients die due to the severity and rapid progression of the disease (2, 3). In HFMD, the most common viral infections are Coxsackievirus A16 (CoxA 16) and Enterovirus 71 (EV71) (4). Due to environmental and demographic factors, Guangdong Province has been identified a high prevalence area for HFMD. Together with influenza and other infectious diarrheal diseases, HFMD is one of the top three Class C infectious diseases in Guangdong Province, making up over 90% of all reported cases (5, 6). HFMD has caused a significant economic burden on families and society, which is one of the major public health issues in Guangdong Province.

Inactivated EV71 vaccination was officially launched in Guangdong Province of China in September 2016. The EV71 inactivated vaccine has been used as a voluntary vaccine, targeting children from 6 months to 3 years (or up to 5 years old). The immunization schedule involves receiving 2 doses of the vaccine (7). Zheng et al. (8) showed that the peak incidence of HFMD in Guangdong Province was dominated by EV71 infections before the launch of the EV71 vaccine. Yang et al. (9) further noted that since the launch of the inactivated EV71 vaccination program, the vaccination coverage in Guangdong Province has gradually increased, and the incidence of EV71-associated HFMD, as well as the number of severe illnesses and deaths decreased in 2017 compared with 2016. An economic evaluation of the EV71 vaccine’s launch in Guangdong Province would be beneficial for reviewing the economic benefits of vaccine’s market and for providing recommendations for existing vaccine policies.

Models are commonly used in the economic evaluation of vaccines. In the economic evaluation of vaccines, models are frequently employed as research tools. The models utilized in the economic evaluation of vaccines based on infectious disease burden can be categorized into static and dynamic models (10). Most of the previous health economic evaluations of EV71 vaccines have been conducted using static modeling, which in practice tends to underestimate the protective effect of the vaccine because they do not account for the indirect protective effect of mass vaccination (11). The relevant guidelines (10) state that dynamic models should be preferred choice for modeling when a particular infectious disease has human-to-human transmission and one of the target populations against that infectious disease is (or includes) a subgroup that can influence the epidemiology of the disease. Many studies have been conducted on the dynamic modeling of HFMD. Typically, these models incorporate additional chambers and adjust parameters to simulate various scenarios (12, 13), such as isolation measures (14, 15), vaccination coverage (16–21), environmental virus clearance rates (22), and hand-washing frequency (16). These simulations aim to evaluate the impact of intervention measures on the prevalence of HFMD. Currently, health economic evaluations of EV71 vaccine interventions predominantly rely on static models (23–27), which are insufficient for assessing the dynamic transmission characteristics of HFMD. Therefore, this study integrates a dynamic model with parameters that more closely align with real-world data to conduct a health economic evaluation of EV71 vaccine interventions, which aims to provide a scientific foundation for public health decision-making in HFMD prevention and control, while also elucidating the economic benefits of vaccination strategies.

## 2 Information and methods

### 2.1 Data sources

Data on the prevalence of EV71 HFMD in Guangdong Province were obtained from the China Disease Prevention and Control Information System, the vaccination rate was obtained from the relevant study conducted by the Guangdong Center for Disease Control and Prevention (GDCCP) (9), and other parameters from the relevant literature.

### 2.2 Research methodology

#### 2.2.1 Model setup

In this study, the year after the start of the vaccine intervention policy (2017) was chosen as the study year. The peak incidence months of that year January to June 2017, were selected as the study timeframe to explore the health and economic benefits of EV71 vaccination for the school-age population (0–5 years old) in Guangdong Province, with the study perspective taken from the patient’s point of view. Based on the SEIR model, this study adopts the method of scenario analysis to evaluate the health economics of EV71 vaccination in Guangdong Province, and four scenarios were constructed: scenario 1 assumes a vaccination rate of 0% (no vaccination); scenario 2 assumes a vaccination rate of 40%; scenario 3, 70%; and scenario 4, 90%. The software used for the study was MATLAB R2023a.

#### 2.2.2 Model description

**Figure 1:**
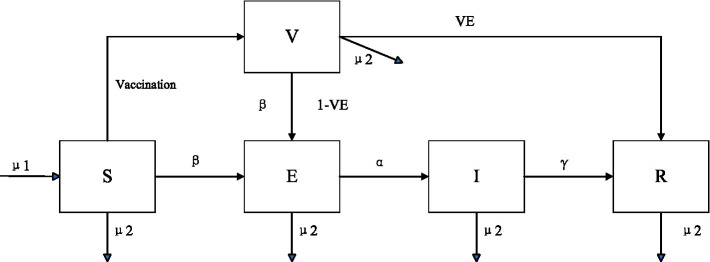
Schematic diagram of the SEIR model of EV71 HFMD.

In this study, based on the natural history of HFMD and vaccination interventions, an extended SEIR model was developed to assess the transmission of EV71 HFMD in Guangdong Province. The population was divided into five bins, with the main populations being susceptible (S), exposed (E), infected (I) and recovered (R), and the vaccinated population (V) was increased according to the vaccination interventions with the parameters of the birth and mortality rates were considered. The total number of people was expressed as N=S + V + E + I + R. The structure of the model is shown in Figure 1, and the model parameters are shown in Table 1.

**Table 1:** Parameters of health economics evaluation of EV71 vaccine.

Parameters	Descriptive	Baseline value	Source (of information etc.)
S(0)	Number of people under 5 years of age in Guangdong Province	2,149,140	Data of the Seven People in Guangdong Province
V(0)	Initial number of vaccinations	82,097	Yang et al. (9)
E(0)	Initial number in latent state	0	RMSE
I(0)	Initial number of infected persons	2,364	RMSE
R(0)	Initial number of people removed from the state	0	Assumed
α	Probability of transition from latent to infected state	0.207	HFMD diagnosis and treatment guideline (2018)
β	Transmission rate (the product of the probability of infection from exposure to an infected person and the rate of exposure)	3.532	RMSE
μ_1_	birth rate	0.01368	Guangdong Provincial Bureau of Statistics
μ_2_	mortality rate	0.0091	Guangdong Provincial Bureau of Statistics
γ	Rate of recovery in the infected population	0.198	HFMD diagnosis and treatment guideline (2018)
ν	Number of vaccinations per month	18,034	Yang et al. (9) Guangdong Provincial Bureau of Statistics
VE	Vaccine efficiency	0.70	Expert consultation
R_0_	Basic regeneration value	8.139	Calculated

*RMSE, root mean square error.

The infectious disease dynamics equations of the SEIR extended model as the following Equations 1–5.


(1) $$S_{n+1}=S_{n}+\mu_{1}N-\frac{{\mathrm{\beta S}}_{n}I_{n}}{N}-\nu -\mu_{2}S_{n}$$

(2) $$V_{n+1}=V_{n}+\nu -\frac{\beta (1-\mathrm{VE})V_{n}I_{n}}{N}-{\mathrm{VEV}}_{n}-\mu_{2}V_{n}$$

(3) $$E_{n+1}=E_{n}+\frac{{\mathrm{\beta S}}_{n}I_{n}}{N}+\frac{\beta (1-\mathrm{VE})V_{n}I_{n}}{N}-{\mathrm{\alpha E}}_{n}-\mu_{2}E_{n}$$

(4) $$I_{n+1}=I_{n}+{\mathrm{\alpha E}}_{n}-{\mathrm{\gamma I}}_{n}-\mu_{2}I_{n}$$

(5) $$R_{n+1}=R_{n}+{\mathrm{\gamma I}}_{n}+{\mathrm{VEV}}_{n}-\mu_{2}R_{n}$$

#### 2.2.3 Model parameters

The monthly positive rate of EV71 HFMD in Guangdong Province from January to June 2017 was obtained from the Guangdong Infectious Disease Reporting System. The number of EV71 cases per month was estimated based on the product of the total number of cases per month and the positive rate of each month. Demographic data for the SEIR model, such as the number of children under 5 years of age in Guangdong Province, the birth rate, and the mortality rate of children under 5 years of age, were obtained from public data provided by the National Bureau of Statistics or the Guangdong Provincial Bureau of Statistics. Parameters such as disease onset time, vaccination rate and vaccine efficacy were derived from relevant studies or expert consultations. Specifically, the initial number of vaccinations and the number of monthly vaccinations were calculated based on the vaccination rates in 2016 and 2017, respectively. According to a survey (9), the average estimated vaccination rate in Guangdong Province was 3.82% in 2016 and 10.07% in 2017. The probability of disease regression was determined based on the estimation of disease incubation time from the latest guideline for HFMD, and the rate of disease recovery was determined based on the estimation of onset time in the guideline (Table 1).

The other parameters in the SEIR model are primarily obtained through fitting processes. The fmincon tool in MATLAB R2023a is utilized to calculate the Root Mean Square Error (RMSE) for random sampling. Through continuous iterations, the parameters are optimized to eventually arrive at the optimal model settings. This tool is designed to solve constrained non-linear optimization problems. The root mean square error is a widely used measure of the discrepancy between predicted and actual values. The smaller the RMSE value, the smaller the difference between the model’s predicted outcomes and the actual observations. The RMSE calculated as follows Equation 6.


(6) $$\text{RMSE}=\sqrt{\frac{1}{n}\sum_{i=1}^{n}{\left(\text{simulated}\_\text{data}-\text{observed}\_\text{data}\right)}^{2}}$$

The above model is based on the following four assumptions:

*Hypothesis 1*: The transmission rates remain unchanged before and after vaccination.

*Hypothesis 2*: Given that the recurrence of HFMD is less likely, a recurrence rate of 0 is assumed.

*Hypothesis 3*: The vaccine’s effect is assumed to be immediate in the population vaccinated against EV71, and vaccination is assumed to have no effect on the epidemiological changes of the dominant pathogens of HFMD.

*Hypothesis 4*: All individuals with EV71 within the study timeframe were children under 5 years of age.

#### 2.2.4 Cost parameters

The study population in this study can be classified into mild outpatient cases, mild hospitalized cases, severe cases and fatal cases according to the clinical classification of HFMD. The relevant incidence rates and number of cases were obtained from the Guangdong Infectious Disease Surveillance System. Treatment cost data for each case type were sourced from the study (28–30) and adjusted to the appropriate year using the China Consumer Price Index for Healthcare. Early death loss is the present value of lifetime earnings (PVLE) lost from the age of death to the expected age for cases that died at a given age. This was done using the human capital method, following the approach outlined in the study by (31). The calculation formula of human capital method is shown in Equation 7. The cost of vaccination was derived from a related report (32), with specific parameter values detailed in Table 2. All costs have been converted to U.S. dollars (USD) using the exchange rate of 1 USD = 6.75 RMB.


(7) $$\text{PVLE}\;\mathrm{per}\;\text{capita}=\sum_{a=s}^{n}\frac{L_{a}\times I_{a}}{{\left(1+r\right)}^{\left(a-s\right)}}$$

**Table 2:** Baseline values and sources of relevant cost parameters.

Cost type	Baseline value	Source (of information etc.)
EV71-related mild HFMD incidence (%)	99.69	Calculated from monitoring data
Proportion of outpatient cases among mild type cases (%)	79.00	Chang et al. (35)
Proportion of inpatient cases among mild type cases (%)	21.00	Chang et al. (35)
EV71-associated HFMD severe disease incidence (%)	0.29	Calculated from monitoring data
EV71-related HFMD mortality incidence (%)	0.02	Calculated from monitoring data
Per capita cost for EV71-related mild type HFMD outpatients	$354.73	Wang et al. (28)
Per capita cost of EV71-related mild type HFMD inpatients	$1,072.00	Zheng et al. (29)
Per capita cost of EV71-related HFMD severe patients	$3,051.00	Zheng et al. (29)
Per capita cost of treating EV71 related HFMD fatal cases	$2,819.00	Zheng et al. (29)
Per capita intangible costs for patients with EV71-associated HFMD	$484.49	Gan (30)
Per capita cost of EV71-associated HFMD patients all lost to early deaths	$164,069.21	Zheng and Yang (31), calculated
Cost of single-dose vaccinations (2017)	$31.26	Guangdong Provincial Center for Disease Control and Prevention (31)

The parameters in the formula are defined as follows:

s is the current age of death of the case;

n is the life expectancy of a person whose age is s;

La is the labor force participation rate at age a; (La = Economically active population/Working-age population, The economically active population refers to the population in the total population of a country or region that has participated in or requires to participate in economic activities, including the unemployed and the employed) Ia is the annual income of people aged a, GDP per capita for each year is used here.

#### 2.2.5 Evaluation methods and indicators

In this study, scenarios 2, 3, and 4 were compared with real-world scenarios in a net benefit analysis. The aim was to assess the economic value of implementing an EV71 vaccination policy in Guangdong Province. The measures of the study included the total number of cumulative infected cases, the overall economic cost, and the net economic benefit. Since the costs included in this paper are mainly direct, indirect and intangible economic costs borne by patients, the study’s perspective is set from the patient’s point of view. Additionally, the time frame of the study in this paper is less than 1 year and does not require discounting.

To gain insight into the economic rationalization of vaccine pricing in 2017, this paper conducts a cost-effectiveness analysis comparing Scenario 1 with a real-world scenario. The evaluation indicators include the cumulative number of infected cases, cost (the cost required for vaccination), benefit (i.e., reduced economic burden), net benefit, and Benefit–Cost Ratio (BCR). The cost primarily refers to the vaccination cost, encompassing both the cost of administering vaccine and the cost of managing adverse reactions. The benefit is the reduction in the economic burden due to vaccination. The net benefit is calculated as the benefit minus the cost. The BCR is the ratio of the benefit to the cost, and is used to measure the economic viability of a policy or project. If the BCR > 1, it means that the policy is economically viable; if not, the economic feasibility is poor. To ensure the reliability of the analysis results, this paper employs single-factor sensitivity analysis in the cost-effectiveness analysis of Scenario 1 and the real-world scenario. This is to examine the impact of different parameter changes on the analysis outcomes, with the range of values for the sensitivity analysis parameters displayed in Table 3.

**Table 3:** Range of parameter values for sensitivity analysis.

Parameters	Scope of change	Maximum value of the parameter	Minimum value of the parameter
Per capita cost for EV71-related mild type HFMD outpatients	±30%	$461.14	$248.31
Per capita cost of EV71-related mild type HFMD inpatients	±30%	$1,433.86	$772.08
Per capita cost of EV71-related HFMD severe patients	±30%	$4,080.89	$2,197.40
Per capita cost of treating EV71 related HFMD fatal cases	±30%	$3,770.56	$2,030.30
Per capita cost of EV71-associated HFMD patients all lost to early deaths	±30%	$213,289.98	$114,848.45
Per capita intangible costs for patients with EV71-associated HFMD	±30%	$629.83	$339.14
Cost of single-dose vaccine(2017)	±30%	$40.64	$21.88

## 3 Findings

### 3.1 Model fitting results

**Figure 2:**
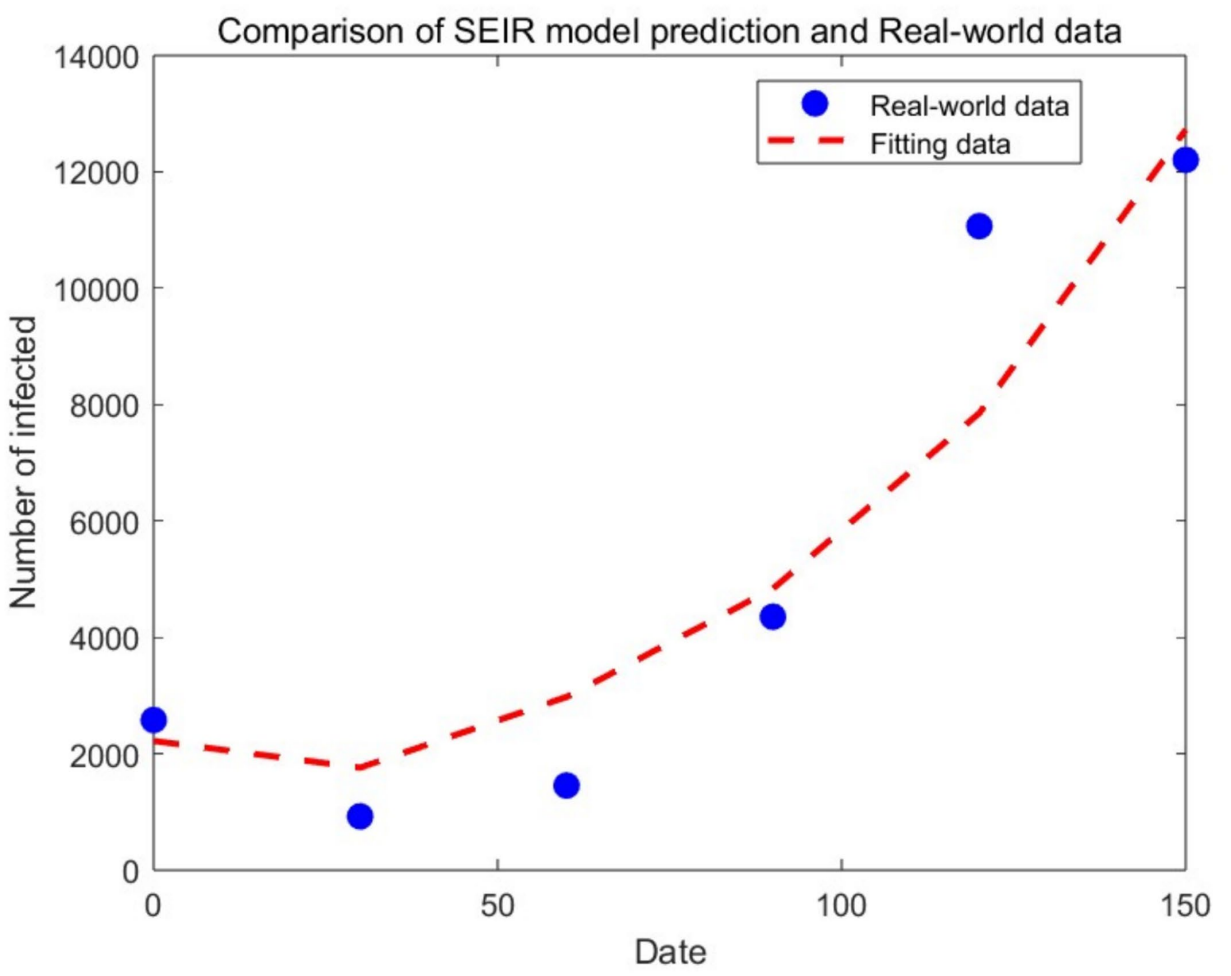
Fitting data based on SEIR model and real-world observed data.

**Figure 3:**
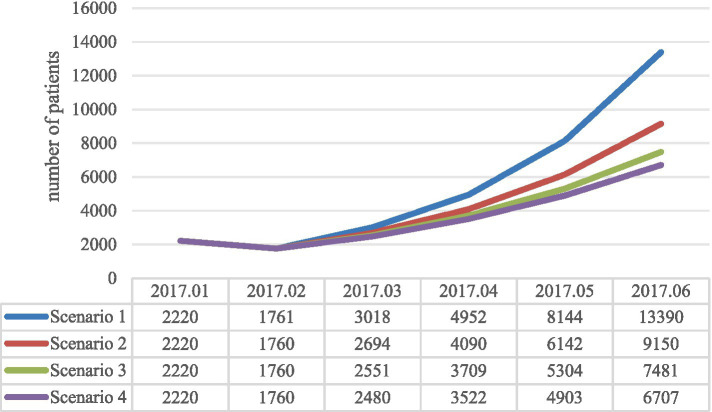
Simulation results of different scenarios based on the SEIR model.

Figure 2 presents the simulation results of comparing the time-monthly number of confirmed cases for Scenario 1 with real-world data, using the actual monthly number of confirmed cases from the first half of 2017 as a benchmark. The results show an *R*^2^ value of 0.997, indicating a close fit with the real-world data and the corresponding basic reproduction number *R*_0_ is 8.139. Figure 3 displays the simulation results under various scenarios. As can be seen from the figure, Scenario 1 (vaccination rate = 0) simulates the highest number of cases with the fastest growth, whereas scenario 4 (vaccination rate = 90%) simulates the lowest number of cases with the slowest growth. The number of EV71 HFMD cases decreases as the vaccination rate increases, with the specific month case numbers for each scenario detailed in Table 4. The cumulative increase/decrease in the number of confirmed cases is the result of a comparison with real-world data. Table 5 presents the composition of the disease types calculated based on the different scenarios and their associated proportions.

**Table 4:** Data on different scenario simulations and real-world cases.

Year/month	The real world	Scenario 1	Scenario 2	Scenario 3	Scenario 4
2017.01	2,579	2,220	2,220	2,220	2,220
2017.02	923	1761	1760	1760	1760
2017.03	1,454	3,018	2,694	2,551	2,480
2017.04	4,354	4,952	4,090	3,709	3,522
2017.05	11,067	8,144	6,142	5,304	4,903
2017.06	12,204	13,390	9,150	7,481	6,707
Cumulative number of confirmed cases	32,581	33,485	26,056	23,025	21,592
Cumulative increase in confirmed diagnoses Number of cases	–	904	–	–	–
Cumulative reduction in confirmed diagnoses Number of cases	–	–	6,525	9,556	10,989

**Table 5:** Disease type composition of the cumulative number of morbidity cases under different scenarios.

Categorization	Confirmed case				Total
	Mild outpatient s	Mild inpatients	Severe cases	Fatal cases	
The real world	25,659	6,821	95	5	32,580
Scenario 1	26,371	7,010	98	6	33,485
Scenario 2	20,520	5,455	76	5	26,056
Scenario 3	18,133	4,820	67	5	23,025
Scenario 4	17,005	4,520	63	4	21,592

### 3.2 Analysis of net benefits

Table 6 reports the results of the net benefit analysis comparing Scenarios 2, 3, and 4 with the real-world scenario. In the real-world scenario, there are approximately 32,580 cumulative cases of disease, with a total economic burden of about $33,540,000. With 40% vaccine coverage, the cumulative number of cases is reduced to 26,056, resulting in a net monetary benefit of about $6,550,000, which represents the reduction in the economic burden of the disease. At 70% vaccine coverage, the cumulative number of morbidity cases is further reduced to 23,025, yielding a net monetary benefit of about $9,590,000. With a 90% vaccine coverage rate, the cumulative number of morbidity cases is reduced to 21,592, with a net monetary benefit of about $11,200,000. It is evident that as the vaccination coverage rate coverage rate increases, the reduction in the cumulative number of EV71 HFMD cases becomes increasing significantly, and the savings in the economic burden of the disease also increase.

**Table 6:** Results of the net benefit analysis for scenarios 2, 3, and 4 versus the real world.

Sight	Cumulative number of incidence cases	Total economic burden (million)	Net monetary benefits (million)
The real world	32,580	$33.54	–
Scenario 2	26,056	$26.99	−$6.55
Scenario 3	23,025	$23.95	−$9.59
Scenario 4	21,592	$22.34	−$11.2

### 3.3 Cost-effectiveness analysis

Table 7 shows the results of the cost-effectiveness analysis comparing Scenario 1 with real-world clearances. If the EV71 vaccine (Scenario 1) is administered at the actual average vaccination rate for the first half of 2017, the benefit to patients, that is, the reduction in the economic burden of the disease, would be approximately $1,080,000. However, it would cost approximately $2,570,000 to achieve that vaccination rate, resulting in a net benefit of approximately −$1,490,000. The cost-effectiveness ratio would be 0.419, which is less than 1. There is no cost-effectiveness advantage from the patient’s perspective and given the current vaccine pricing.

**Table 7:** Results of the cost-effectiveness analysis of Scenario 1 and the real-world scenario.

Sight	Cumulative number of infected cases	Costs (million)	Effectiveness (million)	Net benefit (million)	Incremental cost-effectiveness ratio
Scenario 1	33,485	–	–	–	–
The real world	32,580	$2.57	$1.08	−$1.49	0.419

### 3.4 Sensitivity analysis

**Figure 4:**
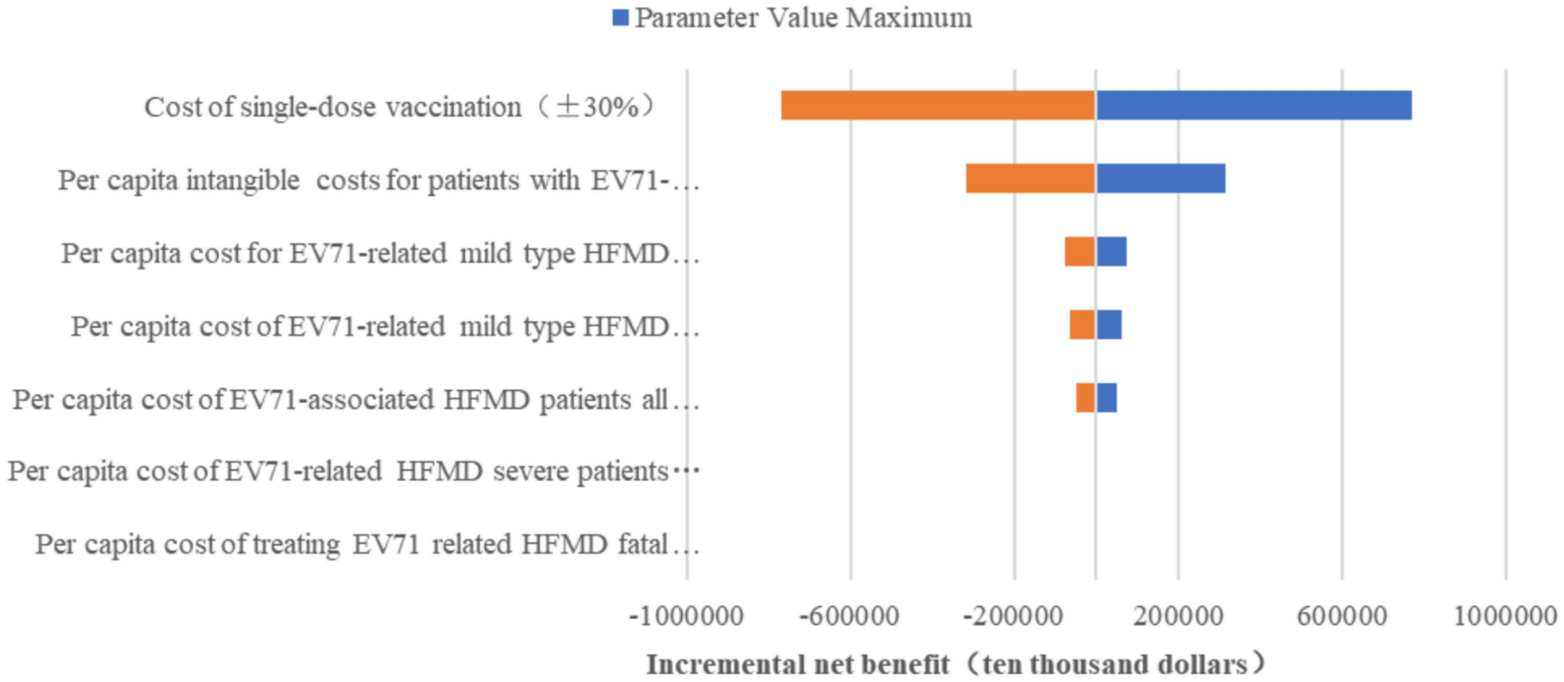
One-way sensitivity analysis.

Figure 4 displays the results of the cyclone diagram of the one-factor sensitivity analysis, indicating that the vaccination cost is the most influential factor affecting the cost-effectiveness outcomes, followed by the intangible costs of EV71 HFMD patients, outpatient costs, hospitalization costs, treatment costs of missed cases and loss due to premature death, in that order. The analysis reveals that the incremental net benefit varies with changes in parameters, except for vaccination costs, without surpassing the baseline net benefit value of $1,490,000. However, when the vaccination costs are reduced by 26.41%, that is, when they are reduced to approximately $13.15 or less, the net benefit becomes positive, and the incremental cost-effectiveness ratio exceeds 1. It is evident that the cost-effectiveness advantage of vaccinating school-age children in Guangdong Province with the EV71 vaccine is realized when the cost of vaccination is less than $13.15.

## 4 Discussion

In this study, we developed a SEIR model with constant model parameters adjusted. According to four scenarios, the EV71 vaccination rate was set to 0% (Scenario 1), 40% (Scenario 2), 70% (Scenario 3), and 90% (Scenario 4). It compared the number of EV71 infections between the vaccinated and the non-vaccinated states as modeled. The aim was to assess the number of cases and the economic burden caused by EV71 in Guangdong Province under different vaccination rates. Finally, a net benefit analysis and cost-effectiveness analysis were conducted.

The results of the infectious disease dynamics modeling show that the basic reproduction value (R0) for EV71 HFMD in the first half of 2017 was approximately 8.139, which is quite close to the results of previous studies (33, 34). Further analysis revealed that the intervention of the EV71 vaccine indeed effectively reduced the number of EV71 HFMD cases during the peak incidence period in 2017. Compared to the scenario without vaccination, the number of EV71 HFMD cases reduced by EV71 vaccination in Guangdong Province from January to June 2017 was about 904 cases. After estimation through the diagnosis rate and consultation rate, the actual number of illnesses reduced was about 2,177 cases, most of them which were mild outpatient cases, non-attendance cases and missed cases. Due to the low vaccination rate in 2017, three additional scenarios were constructed in this study to explore the effect of a high vaccination rate, and it was found that the effect of vaccination on reducing the number of EV71-type illnesses became more and more significant as the vaccination rate increased.

The findings of the health economics evaluation show that in the first half of 2017, Guangdong Province reduced the economic burden of disease by approximately $1,080,000 through the administration of the EV71 vaccine. If the vaccination rate were increased to 40, 70 and 90%, compared with the true vaccination rate, the reduction would be $6,550,000, $9,590,000, and $11,200,000, respectively. The higher the vaccination rate, the more it can help patients reduce the economic burden of disease. Further cost-effectiveness analysis comparing the actual vaccination scenario with a non-vaccination scenario revealed that, from the patient’s perspective, the current vaccine pricing is economically weak. To achieve cost-effectiveness advantages, the vaccine price should be controlled below $13.15.

Our study has several limitations. First, given the possibility that not all hand, foot, and mouth disease (HFMD) cases are registered in surveillance systems, there may be an underestimation of the total number of HFMD cases. Second, individual variations in cost data are likely to exist, leading to discrepancies between real-world data and statistical estimates. To address this issue, we conducted a sensitivity analysis on the cost data. Third, this study utilized conservative coverage data from the onset of the policy, which potentially underestimated the long-term population-level protective effects of the vaccine. Regarding the limitations of the data time frame, we argue that the 2017 data represent the first complete natural year following the introduction of the vaccine in China. Post-2018, multiple interventions in the HFMD prevention and control system, along with changes in the pathogen spectrum of HFMD in Guangdong Province (shifting from EV71 virus dominance to other types due to vaccination), could interfere with independent evaluations of vaccine effectiveness. Furthermore, the outbreak of COVID-19 in 2019 added complexity to the research. During the pandemic, stringent measures such as lockdowns, social distancing, and mask-wearing effectively controlled the spread of infectious diseases, including HFMD, resulting in significant deviations in HFMD incidence rates compared to normal years. Additionally, factors like the reallocation of medical resources and shifts in disease surveillance priorities during the pandemic may have influenced the collection and accuracy of HFMD data, thereby complicating the assessment of the vaccine’s long-term effectiveness.

In summary, the current pricing of the EV71 vaccine is relatively high, and the economic cost is one of the significant factors affecting the public’s willingness to get vaccinated. To address this issue, the government could reduce the cost of vaccination through subsidies, tax concessions by providing other financial incentives to make it more affordable. Additionally, the government could formulate and implementing mandatory vaccination policies, especially by requiring proof of vaccination during critical periods, such as when children are enrolled in childcare and school. Furthermore, encouraging the private sector to participate in vaccine promotion and vaccination efforts and expand vaccination coverage through public-private partnership models.

## Data Availability

The datasets presented in this study can be found in online repositories. The names of the repository/repositories and accession number (s) can be found in the article/supplementary material.
